# Perceptions and attitudes of ICU physicians toward antibiotics prescribing and resistance: A cross-sectional study

**DOI:** 10.1371/journal.pone.0273673

**Published:** 2022-09-15

**Authors:** Esraa Mahrous Shendy, Ahmed A. Elberry, Lamia Hamed Mohamed, Marian S. Boshra

**Affiliations:** 1 Clinical Pharmacy Master Candidate, Faculty of Pharmacy, Beni-Suef University, Beni-Suef, Egypt; 2 Department of Pharmacy Practice, Pharmacy Program, Batterjee Medical College, Jeddah, Saudi Arabia; 3 Department of Clinical Pharmacology, Faculty of Medicine, Beni-Suef University, Beni-Suef, Egypt; 4 Department of Critical Care, Faculty of Medicine, Cairo University, Cairo, Egypt; 5 Department of Clinical Pharmacy, Faculty of Pharmacy, Beni-Suef University, Beni-Suef, Egypt; Bilawal Medical College, Liaquat University of Medical and Health Sciences, PAKISTAN

## Abstract

**Background and aim:**

Antibiotic resistance is a major emphasis in intensive care units (ICUs). Better understanding of ICU physicians’ perceptions, attitudes, and knowledge about antimicrobial prescribing practices could facilitate more effective interventions in fighting antimicrobial resistance in Egyptian ICUs and establishing a proper Antimicrobial Stewardship Program.

**Methods:**

A cross-sectional questionnaire study was conducted including 92 physicians distributed across the different types of Egyptian healthcare institutions in two cities of Egypt; Cairo and El Monufia. Over a period of three months, started in December 2019 and ended in February 2020.

**Results:**

A total of 92 Egyptian physicians were included in the study. Seventy (76.1%) of the surveyed physician strongly agreed and 22 (23.9%) agreed that antibiotic resistance is a worldwide problem. Moreover, 50 (54.3%) strongly agreed and 40 (43.4%) agreed that it is a problem in their hospitals while only 2 (2.1%) disagreed. Poor hand hygiene (67.5%), poor infection control practices by healthcare professionals (63.9%) as well as wrong practices in the management of invasive devices (68.7%), and poor environmental cleaning practices (63.4%) were considered very important causes of AMR by the majority of the surveyed ICU physicians. Almost all of the physicians (95%) rated an advice from a clinical pharmacist as very or moderately helpful intervention, while (52%) declared an advice from a microbiologist or an infectious disease specialist as very helpful.

**Conclusion:**

The results of the present study showed that the Egyptian ICU physicians have remarkable knowledge regarding antibiotic resistance as a worldwide problem and a high sensibility toward the problem in their hospitals. The study also showed that implementation of proper AMS is an urgent need as physicians answers for the different questions in the survey showed that their attitudes and perceptions regarding antibiotic resistance and their way in prescription could be modified and improved if AMS programs with suitable training programs and local guidelines are provided among different types of Egyptian hospitals.

## Introduction

Every day the world is facing new challenges, one of the monumental challenges that face the world nowadays is antimicrobial resistance (AMR) [1]. A recent publication by the Centers for Disease Control and Prevention (CDC) estimated that each year in the U.S., at least 2.8 million people get an antibiotic-resistant infection, from those patients who develop infections, an estimated 35,000 people die each year as a direct result of these infections [2]. Similarly, in Europe, about 25, 000 people die every year from antibiotic-resistant bacteria, Lord Jim O’Neill’s team ‘issued a paper predicting that by 2050, AMR will be responsible for 10 million fatalities per year around the world [3]. COVID-19 is now a new public-health danger [4]. WHO has warned the scientific world that the increased use of antibiotics to combat the COVID-19 pandemic will strengthen the bacterial resistance problem, leading to more deaths during the crisis and beyond [5]. In hospitals, 1450 (72%) of 2,010 COVID-19 patients received broad-spectrum antibiotic therapy, although only 8% of them developed bacterial and fungal co-infections [6]. This unwarranted prescribing pattern could increase the long-term threat of AMR [7].

Antibiotic resistance is a major emphasis in intensive care units (ICUs). ICUs have been described as a factory for developing, propagating, and amplifying antimicrobial resistance [8]. Furthermore, the prevalence of Hospital-acquired infections (HAI) was found to be more common in ICU than in general hospital wards [9]. Along with the problem of HAI goes the burden of Multi-Drug Resistance (MDR). The burden of MDR was related to the high rate of inappropriate empiric broad-spectrum antimicrobial agents [8].

Antibiotics were administered to almost 60% of ICU patients during their stay [10]. Even though only 35% of ICU patients had positive microbiologic cultures, research indicated that 70% of ICU patients were getting at least one antibiotic [11]. Antimicrobial Stewardship Programs (AMS) were described as one of the key interventions to combat AMR [12]. They are a series of interdisciplinary interventions aimed at ensuring the sensible use of antimicrobials by limiting their overuse. These methods can be implemented to help control AMR by raising public awareness and educating healthcare professionals on antimicrobial use especially in low-middle income countries [3].

All the previously stated facts embolden the current study objectives. The survey was conducted to discover ICU physicians’ perceptions and attitudes toward antibiotic prescribing and resistance. In addition to possible ways for combating the problem in Egyptian ICUs, to raise awareness toward antibiotic resistance and changing physician’s behavior for antibiotic prescriptions.

## Material and methods

The current cross-sectional study was conducted across two governorates of Egypt; Cairo and El Monufia. Over 3 months, started December 2019 and ended February 2020. Ethics approval was obtained from the institutional review board of the faculty of Pharmacy, Beni-Seuf University (# Rech ph. bsu 20005). The targeted group was ICU physicians, sample size (n) was calculated using this equation ($n=(Z^{2}*(p)*(1-p))÷c^{2}$) where Z value is 1.96 for 95% confidence leve, (p = 0.5) percentage or proportion is what the results expected to be, confidence interval (c = 0.1) is considered to be the amount of error that can be allowed in the study,(n) was calculated to be 96 physicians.

The completion of the questionnaire by participants was considered an agreement for participation in the study, physicians were insured that data is collected anonymously. Five hospitals of different types were included four of them in Cairo: New Cairo hospital, Kasr ALAiny, El Helal hospital, Railway hospital and International Medical Centre, while one of them in Monufia; Toshiba Al Arabi hospital.

The questionnaire “S1 Table” was adapted from previous studies and extant literature, validity and reproducibility were tested in the cited studies,”S1–S4 Files” [13, 14].

It was pilot tested on a sample of 12 physicians and adjustments were made according to the need and the point of view regarding the study objectives and their data were included. Ninety -two (92) ICU physicians were recruited using the convenient sampling method, they were contacted via mail in which the title, aims, and objectives of the study were clarified in the questionnaire form. Hardcopies were provided for physicians who couldn’t be contacted by mail. The survey was written in English, participation was voluntary, and no incentives for participation were given.

The questionnaire included closed ended questions regarding ICU physicians’ professional profiles, working setting, the existence of antimicrobial stewardship core elements, availability of local guidelines for therapy of infections, infection control team, and availability of periodic reports on local antibiotic resistance data. The questionnaire also included questions regarding physicians’ perception of the importance and causes of antibiotic resistance, their attitudes towards antibiotic prescription, and suggested interventions to improve antibiotic prescription. The clinical pharmacist’s role in antibiotic prescription was also questioned. The answers to questions (Q7-9) varied between (Yes, No, Unsure), while questions about attitudes and perceptions towards antibiotic prescribing and resistance were designed using the 4-point Likert scale with response options from very helpful/important/ confident to very unhelpful/unimportant/unconfident.

### Statistical analysis

After the data were collected and verified, they were coded and entered manually in the excel sheet based on the code given to each hospital data “S1 Dataset”. It was analyzed using Statistical Package for the Social Sciences (SPSS) program version 25.

The chosen statistical test was chi-square. The data were expressed as frequency and percentage. The confidence level was 95%. The significance was evaluated by comparing the actual value against a critical value found in a Chi-Square distribution, p-value ≤ (0.05) was considered significant.

## Results

### Baseline characteristics, hospital profile, and hospital’s antimicrobial stewardship core elements

Surveyed ICU physicians’ baseline characteristics, hospital profile, and antimicrobial stewardship core elements are described in “Table 1”.

**Table 1:** Baseline characteristics of the participants, hospital profiles and antimicrobial stewardship core elements.

• **Baseline Characteristics**	**N (%)**
1. Gender	
Male	69(75)
Female	23 (25)
** 2. Years of experience**	
Less than 10 years	67 (72.8)
10–20 years	21 (22.8)
21–30 years	4 (4.3)
• **Hospital profile:**	
** 3. Type of the hospital**	
University Hospital	36 (39.1)
Private Hospital	32 (34.8)
Ministry Hospital	18 (19.6)
Other (e.g., military hospitals)	4 (4.3)
All	2 (2.2)
**4. Hospital inpatient beds**	
Less than 100	29 (31.5)
100–500	39 (42.4)
501–1000	9 (9.8)
More than 1000	8 (8.7)
Unsure	7 (7.6)
**5. Hospital ICU beds**	
5–10	12 (13.0)
11–20	27 (29.3)
21–30	21 (22.8)
More than 30	32 (34.8)
**6. Type of the ICU**	
Surgical	22 (23.9)
Medical	58 (63.0)
Neurology	3 (3.3)
Cardiology	6 (6.5)
Other (e.g., pediatric)	3 (3.3)
• **Antimicrobial stewardship core elements**	
**7. Availability of antimicrobial stewardship in the hospital**	
Yes	61 (66.3)
No	21 (22.8)
Unsure	10 (10.9)
**8. Availability of local guidelines for therapy of infection in the hospital**	
Yes	68 (73.9)
No	19 (20.7)
Unsure	5 (5.4)
**9. Periodical departmental reports on local antibiotic resistance data**	
Yes	64 (69.6)
No	23 (25)
Unsure	5 (5.4)

### Participants’ perceptions of the importance and causes of the antibiotic resistance problem

Seventy (76.1%) of the surveyed physicians strongly agreed and 22 (23.9%) agreed that antibiotic resistance is a worldwide problem. Moreover, 50 (54.3%) strongly agreed and 40 (43.4%) agreed that it is a problem in their hospitals while only 2 (2.1%) disagreed.

There was no significant association between physicians’ years of experience (P = 0.977), type of hospital (P = 0.707), and type of ICU (P = 0.434), and their agreement that antibiotic resistance is a worldwide problem. Yet their consideration of antimicrobial resistance (AMR) as a problem in their hospital was affected by the type of hospital, where physicians working in university hospitals (P = 0.020) had a significant p-value < 0.05 compared to other types of hospitals (Private, Ministry, Military). In addition physicians working in medical ICU (P = 0.01), had significant p -value < 0.05 compared to (Surgical, Neurology, Cardiology, Pediatric).

On the other hand, it wasn’t affected by years of experience (P = 0.276).

**Fig 1:**
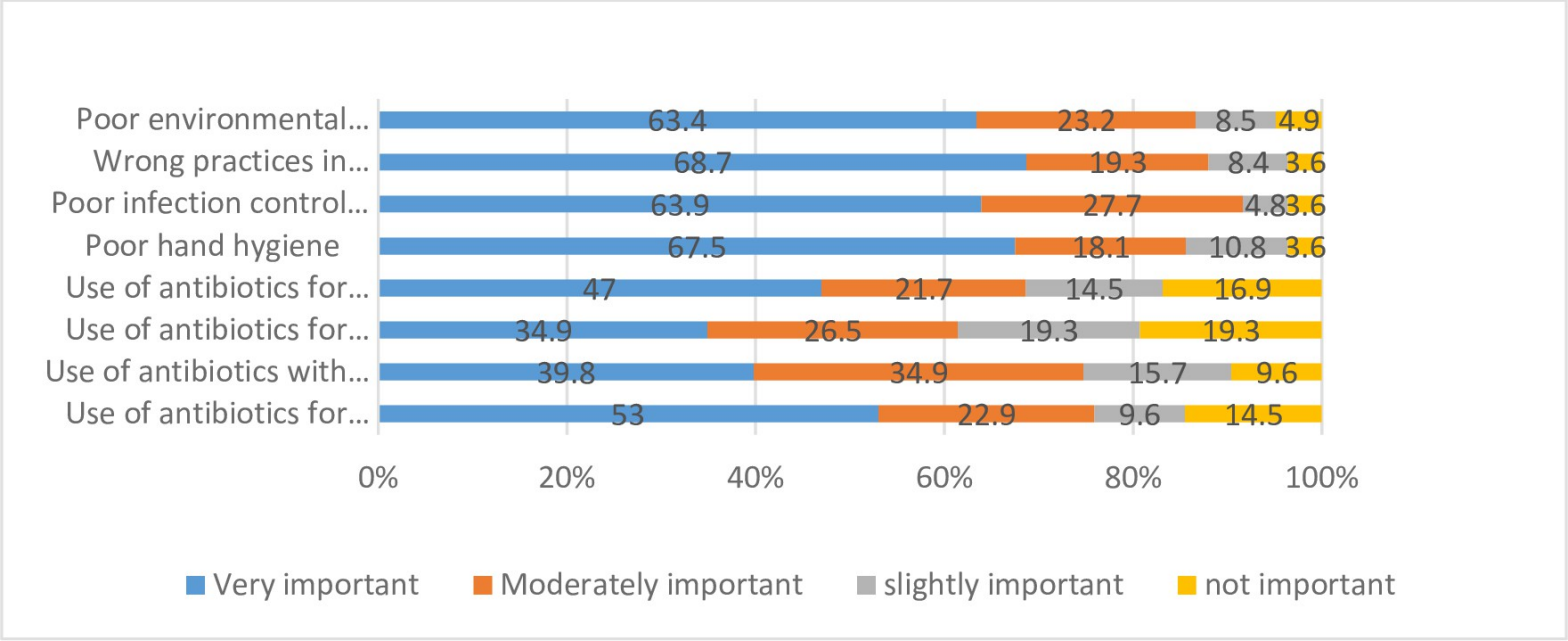
Perception of physicians regarding contributing factors to the development or spread of antimicrobial resistance.

**Fig 2:**
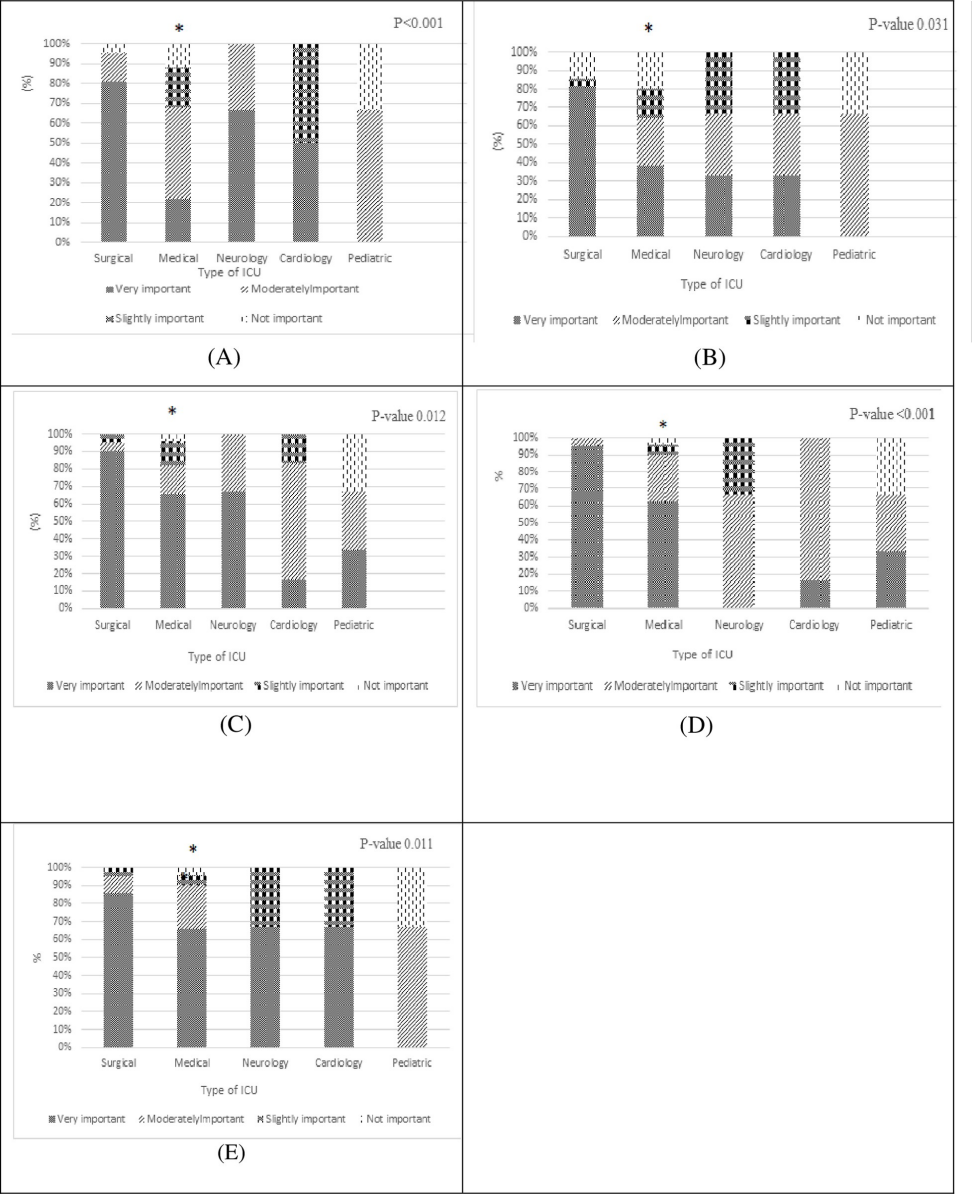
Relationship between physician’s perceptions regarding causes of AMR and type of ICU (* significant p -value < 0.05 compared to other types of ICUs (Surgical, Neurology, Cardiology, Pediatric)). (A) shows perception of physicians regarding using broader than necessary spectrum. (B) shows perception of physicians regarding the cause (shorter than standard duration). (C) shows perception of physicians regarding the cause (poor hand hygiene). (D) shows perception of physicians regarding the cause (poor infection control). (E) Perception of physicians regarding the cause (wrong practices in management of invasive devices).

Physicians were asked to share their perceptions of causes of AMR, their answers were reported as (%) in “Fig 1”. Neither Physician’s years of experience nor type of hospital affected physicians’ answers regarding their perceptions toward contributing factors of AMR. In contrast, there was a significant difference between the physician’s answers working in different ICUs, “Fig 2”.

Additionally, almost all of the surveyed physicians thought that the presence of an infection control team is highly important (61%), moderately important (29%), slightly important (7%) in their hospitals while only (3%) answered it was not important. Unfortunately, physicians answered that their patients are highly likely (41.3%) to suffer from hospital acquired infections, and (50%) answered likely, and (8.7%) answered it’s unlikely.

### Participants’ attitudes and factors influencing the antibiotic prescribing process

**Fig 3:**
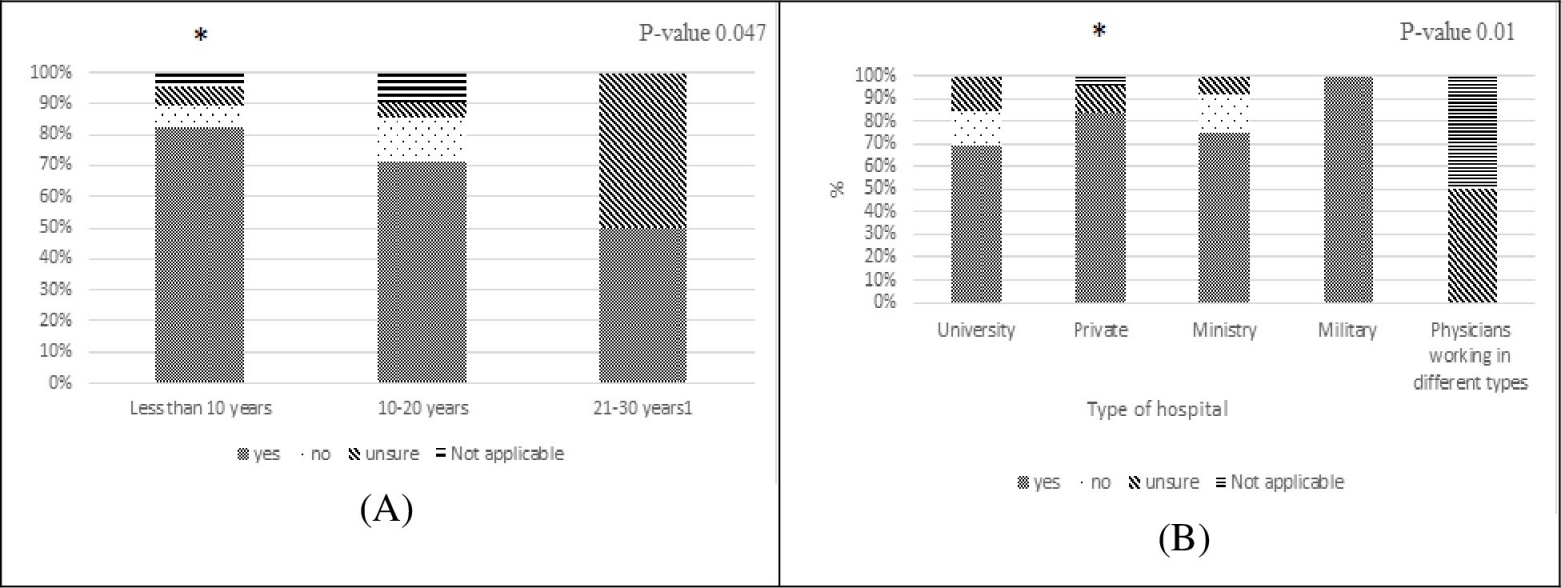
(A) The significant relation between physicians’ years of experience (<10 years) and their consultation to the international guidelines. (* significant p -value < 0.05 compared to more experienced physicians). (B) The significant relation between physicians working in private hospitals answer’s and their consultation to the local guidelines (* significant p -value < 0.05 compared to other types of hospitals (University, Ministry, Military).

Physicians with less than 10 years of experience checked international guidelines more than the other categories (p = 0.047), while the consultation of the local guidelines in their last month was affected by the type of hospital.The physicians who worked in private hospitals (P value = 0.010) tended to consult the local guidelines more than the other categories, “Fig 3”. The majority of the physicians (90.3%) thought that locally developed guidelines for antibiotic treatment are more useful than international ones.

**Fig 4:**
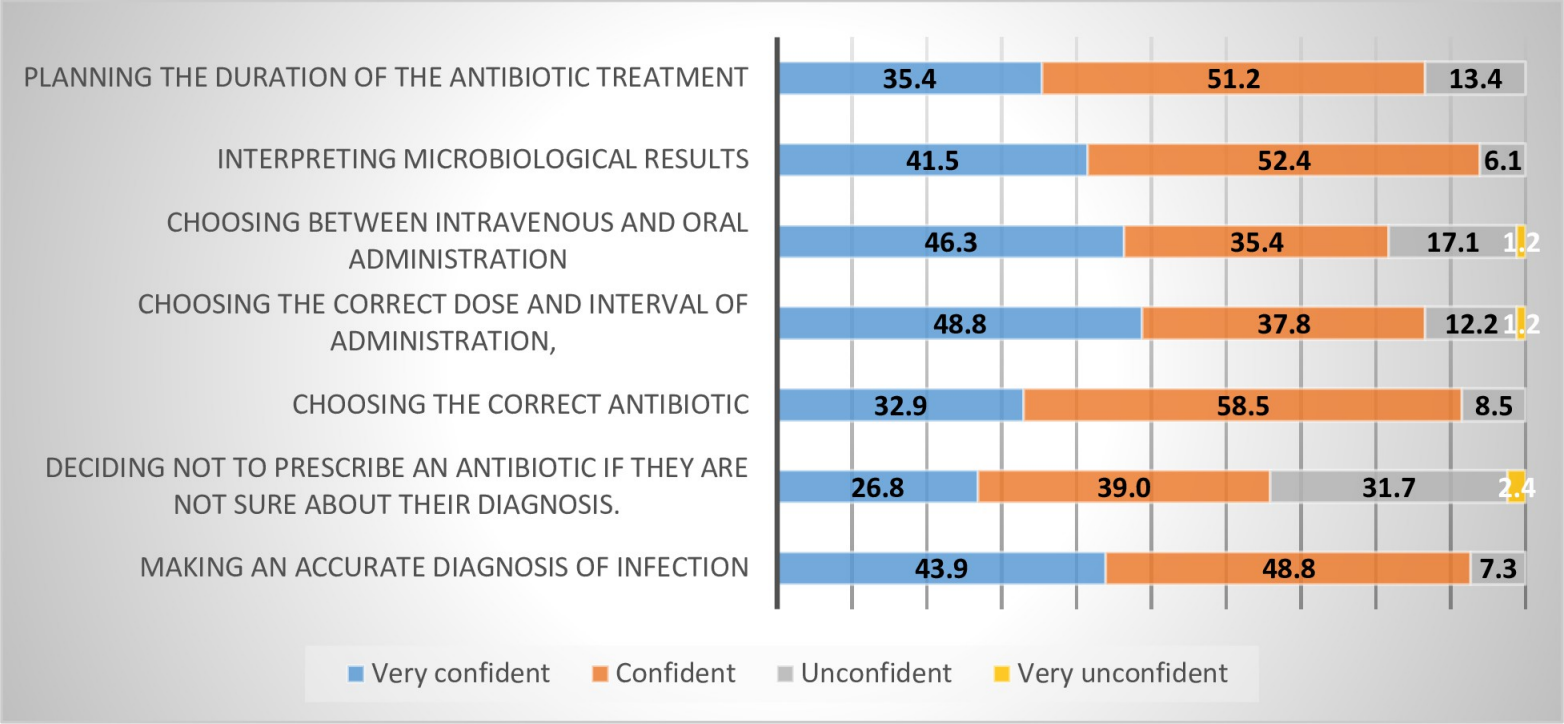
Level of confidence in prescribing the antibiotics, data presented in (%) (n = 92).

The surveyed physicians were asked considering their daily activities how confident did they feel in the following scenarios when they prescribed an antibiotic “Fig 4”.

**Fig 5:**
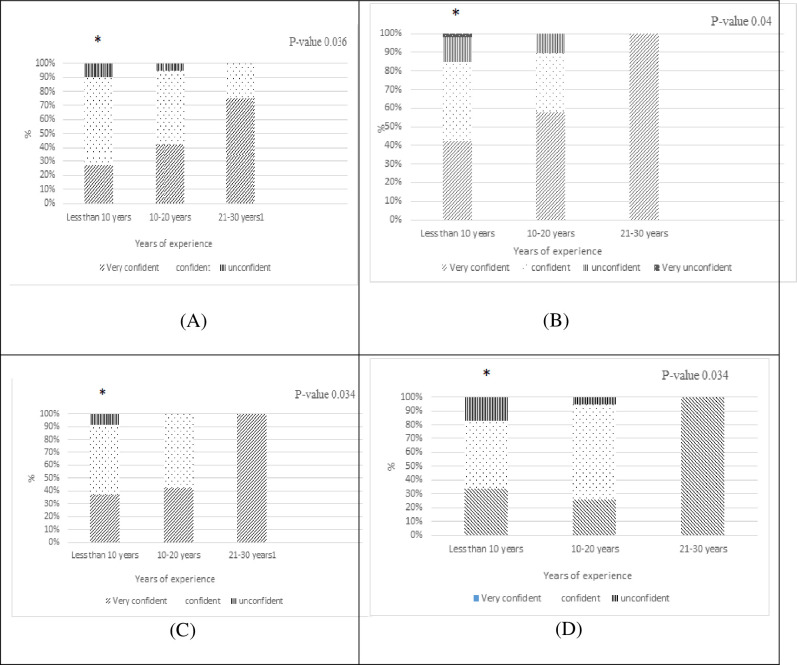
Significant relationship between physician’s years of experience and level of confidence of prescribing antibiotics in various scenarios, (* significant p -value < 0.05 compared to more experienced physicians). (A) choosing the correct antibiotic scenario. (B) choosing the correct dose and interval of administration scenario.(C) interpreting results. (D) Planning the duration of the antibiotic treatment.

Whereas physicians with less than 10 years of experience declared to be more confident to prescribe antibiotics when compared to more experienced physicians in choosing the correct antibiotic, choosing the correct dose, and interval of administration, interpreting microbiological results, planning the duration of antibiotic treatment, “Fig 5”.

In interpreting the microbiological results scenario, physicians’ answers were significantly different with the type of ICU. Physicians working in medical ICU tended to be more confident when prescribing antibiotics in this scenario (p- value <0.001). Physician’s answers working in hospitals with AMS programs were significantly different, they tended to be confident to prescribe an antibiotic when making an accurate diagnosis of antibiotic treatment (p-value 0.007).

Only 31(33.7%) of the surveyed physicians had attended formal training in antibiotic prescribing in the last 12 months while 56 (60.8%) didn’t attend any training additionally 5 (5.5%) were unsure, but 80 (87%) answered that they would like to attend a training in the future, 9 (9.8%) didn’t like to attend any and 3 (3.2%) were unsure.

### Participant’s perceptions of the helpfulness of potential interventions to improve antibiotic prescribing

**Fig 6:**
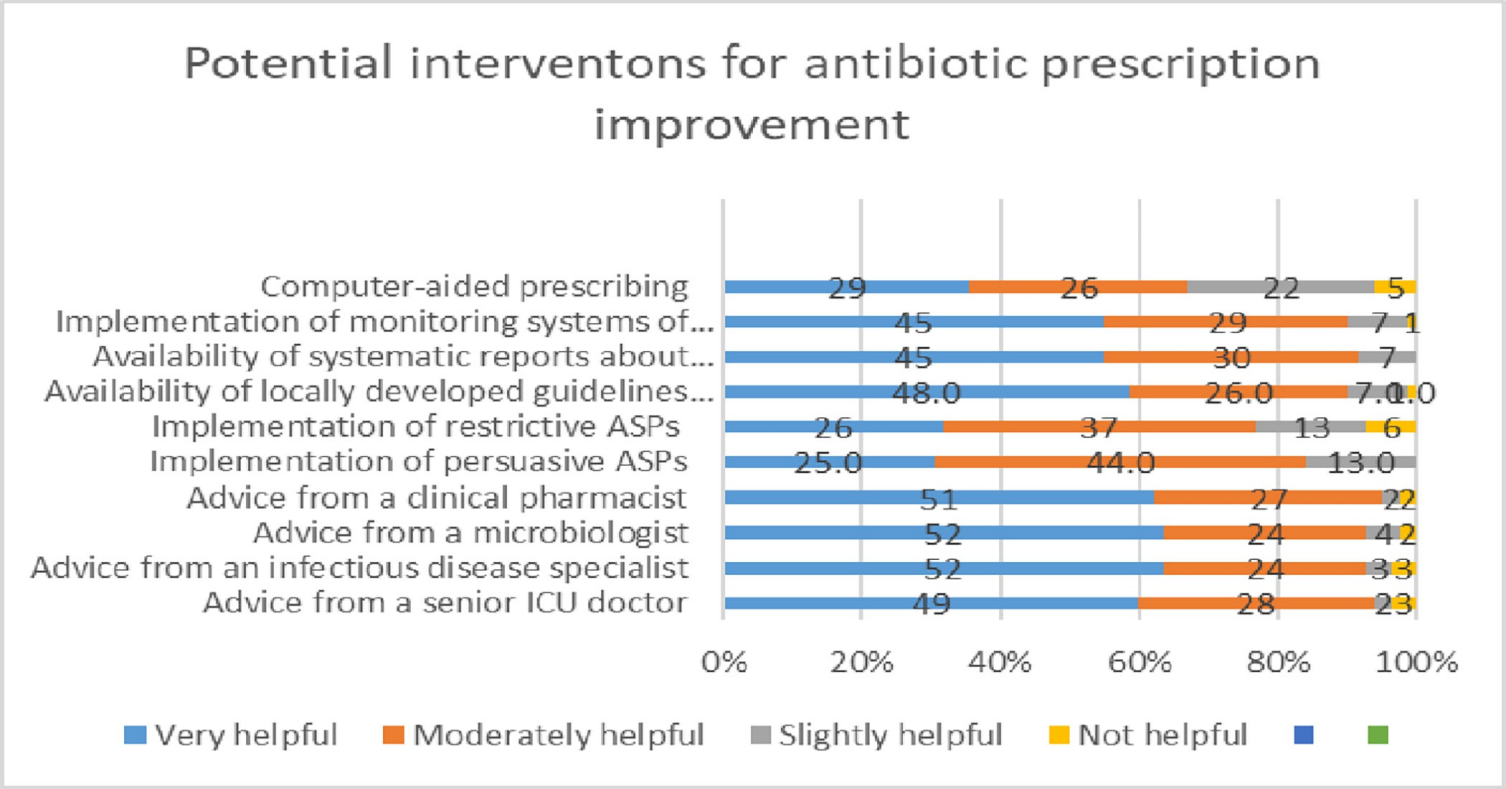
Potential interventions for improvement of antibiotic prescription, answers represented as (%).

**Fig 7:**
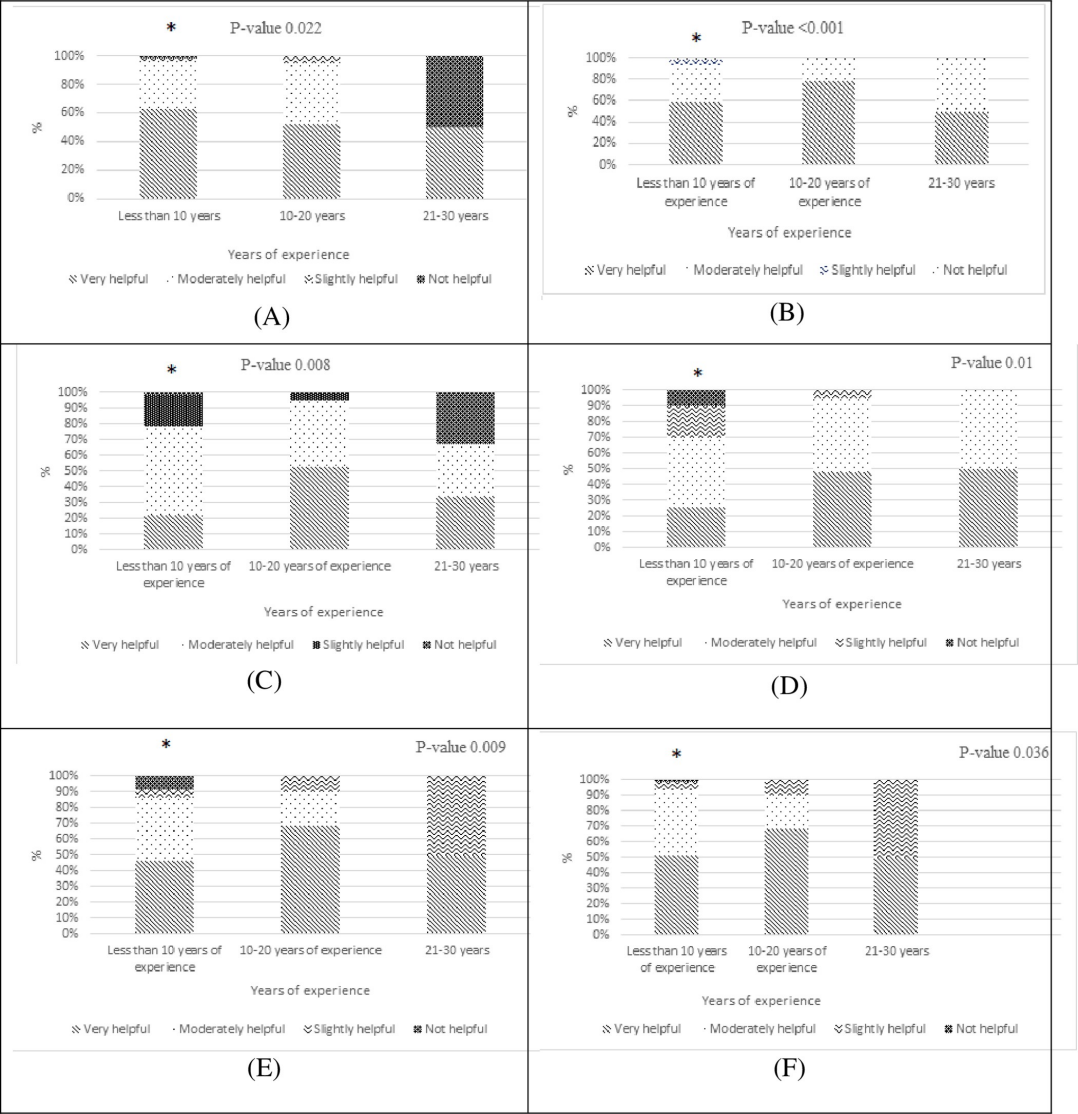
Significant relationship between physician’s years of experience and the following interventions (* significant p -value < 0.05 compared to more experienced physicians). (A) Advice from ICU senior doctor, (B) Advice from an infectious doctor, (C) Implementation of persuasive ASP, (D) Implementation of restrictive ASP (E) Availability of systematic reports about resistance, (F) Implementation of monitoring systems of used antibiotics.

Physicians’ ratings of the helpfulness of potential interventions to improve antibiotic prescribing were reported in “Fig 6”. Physicians with less than 10 years of experience answers were significantly different, they thought that the following interventions were very helpful, “Fig 7”; advice from a senior ICU doctor (0.022), advice from an infectious disease specialist (<0.001), implementation of persuasive ASPs (0.008), implementation of restrictive ASPs (0.01), availability of systematic reports about resistance data (0.009, implementation of monitoring systems of used antibiotics (0.036).

The physician’s answers working in university hospitals were significantly different **compared to other types of hospitals; private, ministry, military** regarding the implementation of monitoring systems of used antibiotics (p -value 0.007).

**Fig 8:**
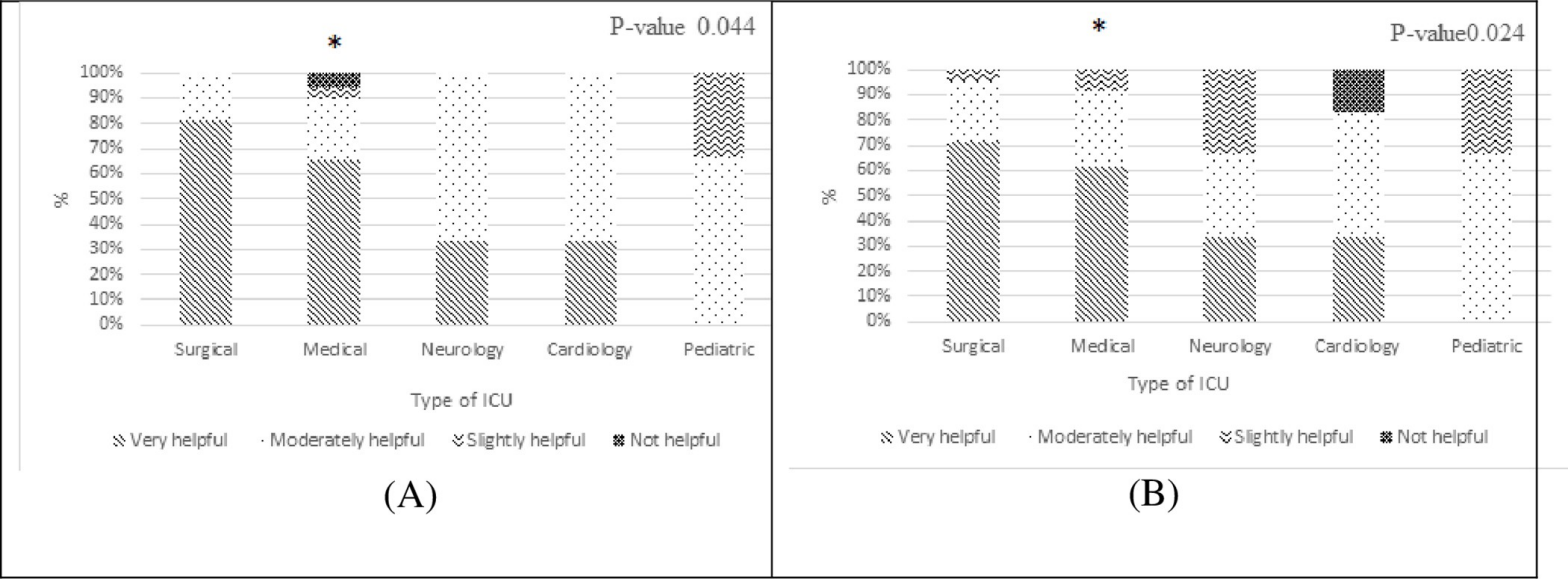
The significant relationship between type of ICU and the following interventions (A) of an advice from an infectious disease specialist (B) availability of locally developed guidelines for therapy of infections intervention. (* significant p -value < 0.05 compared to surgical, neurology, cardiology, pediatric).

Physicians answer’s working in medical ICUs were significantly different, “Fig 8”. They have chosen having advice from an infectious disease specialist (P -value 0.044), and the availability of locally developed guidelines for therapy of infections (P-value 0.024) as helpful interventions.

All of the physicians (94.6%) appreciated the usefulness of the presence of a clinical pharmacist during the ICUs round. About one third of them (33.7%) consulted the clinical pharmacist 10 times monthly regarding choosing antibiotics.

## Discussion

Previous studies were done to assess physicians’ perceptions about antibiotic resistance, in low -middle income countries [15, 16]. The present survey showed that intensive care physicians in Egypt show a great knowledge of the antibiotic resistance problem. The physicians’ knowledge regarding antibiotic resistance varied in correlation with many factors; their years of experience, type of hospital, type of ICU, also the availability of stewardship core elements in the facility.

The majority of the surveyed physician agreed that antibiotic resistance is a worldwide problem. Moreover, more than 50% of physicians agreed that AMR is a problem in their hospitals this was similar to the findings of a previous study done in Fayoum government [17]. On the other hand, this was opposite to what was found by another studies, where physicians approved that the problem is worldwide but underrated the problem in their hospitals, perceiving the risk as more theoretical than the real one [14, 18]. This could be due to insufficient monitoring of multidrug-resistant organisms or a lack of data sharing.

The majority of physicians stated that poor hand hygiene is one of the major causes of AMR. This has been previously mentioned in a previous study which have stated that cross-infection of patients by healthcareworkers using contaminated hands could be a significant source of infections [19]. In addition, a study carried out in Zagazig university hospitals, has reported that lack of compliance by healthcare providers with hand hygiene measures, and environmental contamination were the most common causes of infection in intensive care units [20]. Those findings were similar to multiple surveillance studies conducted in Europe, the United States, Egypt, and Saudi Arabia [21–23]. Studies have shown that improvements in hand hygiene are associated with lower healthcare-associated infection rates, a reductions in Multi-drug resistant organisms (MDRO) transmission and acquisition, a decrease in the use of broad-spectrum antibiotics and finally reduction in antibiotic resistance [24, 25].

Wrong practices in the management of invasive devices as a cause for AMR have been reported by recent surveillance carried out in a medical-surgical ICU from a large tertiary care hospital of Cairo University in Egypt. High incidence rates of Device Associated -Hospital Acquired Infections (DA-HAIs) together with the high levels of antimicrobial resistance pattern were found [26]. This spotlight on the role that the infection control team could play as an intervention in decreasing the level of antibiotic resistance in Egypt’s intensive care units. Campaigns that promote awareness should be encouraged such as "Your 5 Moments for Hand Hygiene “which is recommended by WHO, educational programs regarding proper care bundles such as Catheter associated urinary tract infection (CA-UTI), ventilator-associated pneumonia (VAP), central line-associated bloodstream infection (CLABSI), and surgical site infection (SSI). This was mentioned before in a study done in Egypt where the incidence rate of CA-UTI, VAP,CLABSI were higher than the rates reported in figures of the Annual Epidemiological Report, 2016 of the European Centre for Disease Prevention and Control [20].

A small number of participated physicians attended formal training in antibiotic prescription during the last year. This can be attributed to their workload that sometimes didn’t permit them to attend lectures and training programs as there is a conflict between work and training time. This was a challenge for a previous study done to train physicians before establishing the AMS program [27]. In addition, a previous study proved that the lack of proper training or education of healthcare providers regarding proper antimicrobial use in LMIC is one of the most important factors affecting antimicrobial resistance [15].

Physicians were very confident to prescribe an antibiotic in the following scenarios, choosing the correct dose and interval of administration and choosing between intravenous besides oral administration, and confident when choosing the correct antibiotic and interpreting microbiological results, those findings are consistent with a previous study done on final-year medical students across European medical schools [28]. In contrast to the expected and stated by previous studies in other countries, physicians were unconfident in deciding not to prescribe an antibiotic if they are not sure about the diagnosis, this may occur due to anxiety to miss infection rather than using unnecessary antibiotics and that the more is better [16].

The physician’s years of experience affected the physician’s answers, physicians with less than 10 years of experience were more confident, this is in contrast to a previous study done in Ghanaian tertiary care hospitals which stated that senior physicians had a better knowledge rather than junior ones [29]. However our finding is consistent with another studies which found no association with previous experience, training level, and knowledge of physician [13, 17]. This is supported in our study by finding no difference in the confidence level of physicians who previously received formal training from who didn’t. This can reflect a defect in the training program that should be properly designed to provide physicians with the needed knowledge regarding the antibiotic prescription [30].

Physicians were asked to assess the helpfulness of potential interventions to improve antibiotic prescribing. About 95% have chosen taking advice from a clinical pharmacist to be a very or moderately helpful intervention. This was a promising answer, as a previous study has found that the rate of acceptance of the critical care physicians to the pharmacists’ recommendations, has been estimated to be the lowest in comparison with other departments [31]. Hence the present study focused on the role that clinical pharmacists should play in auditing and rationalizing antibiotic use, and that this information should be emphasized for the junior ICU physicians to ensure harmony between the two teams.

In contrast to previously available studies where less support was found to interventions that restrict physicians autonomy and complicate antibiotic order [32]. In the current study the implementation of persuasive and restrictive ASPs was rated as very or moderately helpful intervention by 88.4% and 82.7% by the physicians in hospitals that have antimicrobial stewardship team. While around 90% of the physicians who are working at departments that periodically receive reports on local antibiotic resistance data rated the availability of systemic reports on resistance as very or moderately helpful. This was confirmed in a previous study that has shown the importance of local resistance reports on perceptions of physicians. It can be used not only to assess trends in antibiotic resistance rates but also to educate clinicians on the proper use of antibiotics [14]. Those choices were significant for less experienced physicians.

The majority of the physicians thought that locally developed guidelines for antibiotic treatment on the institutional level are more useful than international ones. This was traced back to the fact that those guidelines are tailored by the AMS program based on the site of infection, the common pathogens suspected, local epidemiology data, and resistance patterns in the facility. Unfortunately those guidelines are not highly common in low income countries as AMS program are still rudimentary [12, 33]. This finding was opposed by another study done in Egypt where physicians tended to think that international guidelines are more useful than a national one [15].

Age seemed to affect the physician’s answer, where physicians with less than 10 years of experience were the highest category to consult local guidelines, this could be explained by the fact that senior doctors always rely on their clinical knowledge and experience to guide their antimicrobial prescribing practice, and they frequently consider their patients to be "outside" the boundaries of local evidence-based infection treatment policies [34].

There was no significant difference in answers of physicians working in hospitals with or without the AMS program which proves that the present AMS program is still rudimentary and more collaboration is needed between different stakeholdersl [35].

## Conclusion

This survey conducted on Egyptian ICU physicians has shown their remarkable knowledge and high sensibility toward antibiotic resistance as a worldwide problem. The study has shown that implementation of proper infection control & AMS in LMICs is an urgent need. AMS programs should include local guidelines implementation as well as proper training programs about antibiotic prescription. The type of hospital, ICU, and years of experience affected physician’s perceptions regarding antibiotic resistance, all those factors should be taken in consideration when establishing AMS programs.

## Limitation

1. The sample size and sampling method: convenient sampling method was used; the sample wasn’t big enough to generalize the data on all Egyptian hospitals. This sample size and method is due to the limited number of critical care specialty as Egypt faces an unprecedented wave of emigration by physicians Over the past three years, more than 10,000 doctors have left the country, according to the main association representing physicians, the Egyptian Medical Syndicate. The syndicate estimates that half the country’s doctors, or 110,000 out of 220,000 registered doctors, have left the country [36].

### Point of strength

To our knowledge, this is the only Egyptian survey to be done on a specific specialty like intensive care unit.

### Research gap

In the future, it is recommended to conduct a survey with a large sample size to assess physicians’ attitudes and perceptions regarding antibiotic resistance post implementation of ASP in different types of Egyptian hospitals and post proper training of physicians.

## Supporting information

S1 Table (DOCX)

S1 Dataset (XLSX)

S1 List List of abbreviations. (DOCX)

S1 File Factor loadings from principal components analysis with varimax rotation and Cronbach alpha for each domain. (DOCX)

S2 File Test-retest of the domains: consistency two way mixed single ICC. (DOCX)

S3 File Mean and standard deviation (SD) of each domain by surgeons’ professional profile and working setting (validation sample). (DOCX)

S4 File Mean and standard deviation (SD) of each domain by surgeons’ professional profile and working setting (final sample). (DOCX)
