# Supplementary material for: Perceptions and attitudes of ICU physicians toward antibiotics prescribing and resistance: A cross-sectional study
Source: PLoS One. 2022 Sep 15;17(9):e0273673. doi: 10.1371/journal.pone.0273673 (PMC9477304; doi:10.1371/journal.pone.0273673)
Supplement: S1 Table — (DOCX) [file pone.0273673.s001.docx]

**S1 Table**

| 1. **Physician professional profile represented in Question (1-3), Question (6*)***   1.Gender  2.How many years of residency training experience do you have as an ICU doctor?  3.What type of hospital are you working in? If you are working in more than one hospital, please take into account the one where you practice the most of your time.    6.What type of ICU do you mostly work at?   1. **Working setting characteristics (Questions 4-5)**   4.How many inpatient beds has the hospital where you practice the most of your time?  5. How many ICU beds has the hospital where you practice the most of your time?   1. **Antimicrobial stewardship core elements availability (Questions 7-9)**   7.Does your hospital have an antimicrobial stewardship team?  8.Does your Department have local guidelines for therapy of infections?  9.Does your Department periodically receive reports on local antibiotic resistance data?       1. **Participants’ perceptions of the importance of the problem of antibiotic resistance (Question 10-11)**   10.Do you think that antibiotic resistance is a worldwide problem?  11. Do you think that antibiotic resistance is a problem in your hospital?     1. **Participants perceptions regarding Causes of Antimicrobial resistance (Q 12)**   12. Tell us your perceptions for each of the following statements regarding their relevance as contributing factors to the development or spread of antimicrobial resistance:     \| 1. Use of antibiotics for self-limited non bacterial infections (e.g. viral meningitis ) \| \| --- \| \| 1. Use of antibiotics with a broader-than-necessary spectrum \| \| 1. Use of antibiotics for longer than standard duration \| \| 1. Use of antibiotics for shorter than standard duration \| \| 1. Poor hand hygiene \| \| f. Poor infection control practices by healthcare professionals  g. Wrong practices in management of invasive devices  h. Poor environmental cleaning practices   1. **Perceptions regarding infection control (Q 13-14)**   13.How do you see the presence of an infection control team in your hospital?  14. Do your patients likely suffer from Hospital Acquired Infections?     1. **Physician’s and their colleague’s prescription attitude (Q 15-16)**   15. Do you think that your antibiotic prescriptions contribute to the problem of antibiotic  resistance**?**  16.Do you think that your colleagues’ prescriptions contribute to the problem of antibiotic  Resistance?   1. **Physicians future expectations for antibiotic resistance (Q 17-18)**   17. Do you expect that antibiotic resistance will be a greater clinical problem for your patient’s in  the future?  18. Do you expect that new antibiotics will be developed in the next 10 years will keep up with  the problem of resistance?   1. **Physicians’ last month antibiotic prescribing practice (Q 19-21)**   **Answers choices were (Yes-No-Unsure-Not applicable)**   1. In the last month, have you personally used or consulted local guidelines for therapy of infections when considering an antibiotic for a patient**?** 2. In the last month, have you personally used or consulted international guidelines for therapy of infections when considering an antibiotic for a patient? 3. In the last month, have you personally consulted reports on local resistance data to select an antibiotic empiric therapy for a patient? 4. **Physician’s perceptions of factors influencing the antibiotic prescribing process (Q 22)** 5. Considering your daily clinical activities at the moment, how confident do you feel in the following scenarios when prescribing an antibiotic?  \| 1. Making an accurate diagnosis of infection \| \| --- \| \| b. Deciding not to prescribe an antibiotic if you are not sure about your diagnosis \| \| 1. choosing the correct antibiotic \| \| 1. Choosing between intravenous and oral administration 2. Interpreting microbiological results 3. Planning the duration of the antibiotic treatment  \|  \| \| --- \| \| 1. **Formal training in antibiotic prescription (Q 23-24 )** \| \| \| 1. In the last 12 months, have you received formal training in antibiotic prescribing? 2. Would you like to receive more training in antibiotic prescribing in your hospital? \| \|  \| \|  \|  1. **Perceptions of the helpfulness of potential interventions to improve antibiotic**   **Prescribing (Q 25)**  25.Please evaluate, in your opinion, the helpfulness of the following measures to improve  antibiotic prescribing at the moment:   \| 1. Advice from a senior ICU doctor \| \| --- \| \| 1. Advice from an infectious disease specialist \| \| 1. Advice from a microbiologist \| \| 1. Advice from a clinical pharmacist \| \| 1. Implementation of persuasive ASPs \| \| 1. Implementation of restrictive ASPs \| \| 1. Availability of locally developed guidelines for therapy of infections \| \| 1. Availability of systematic reports about resistance data \| \| 1. Implementation of monitoring systems of used antibiotics \| \| 1. Computer-aided prescribing \|  1. **Clinical pharmacist role in antibiotic resistance problem (Q26-28)** \| \| 26.Do you find the presence of clinical pharmacist useful during the ICUs round?    27.How often do you contact the clinical pharmacist regarding choosing antibiotics?   1. In what situations do you find clinical pharmacist important  - Choosing empiric antibiotic post admission - Failure of response to an ongoing antibiotic - Choosing antibiotic in cultures with resistant bacterial species - Immunocompromised patients (e.g. oncology patients) - All of the previous  1. **Comparison of local developed guidelines and international guidelines (Q29)** 2. Do you think locally developed guidelines for antibiotic treatment are more useful than international ones? \| \|  \| |
| --- | --- | --- | --- | --- | --- | --- | --- | --- | --- | --- | --- | --- | --- | --- | --- | --- | --- | --- | --- | --- | --- | --- | --- | --- | --- | --- | --- |
