# Supplementary material for: Perceptions and attitudes of ICU physicians toward antibiotics prescribing and resistance: A cross-sectional study
Source: PLoS One. 2022 Sep 15;17(9):e0273673. doi: 10.1371/journal.pone.0273673 (PMC9477304; doi:10.1371/journal.pone.0273673)
Supplement: S1 List — (DOCX) [file pone.0273673.s003.docx]

| ICU    AMR  CDC  WHO  HAI    MDR  SPSS  ASP  MDRO  DA-HAIs  CA-UTI  VAP  CLABSI    LMIC | Intensive Care Unit  Antimicrobial resistance    Centers for Disease Control and Prevention  World Health Organization  Hospital acquired infections  Multi Drug Resistance  Statistical Package for the Social Sciences  Antimicrobial stewardship program  Multi-drug resistant organisms  Device Associated -Hospital Acquired Infections  Catheter associated urinary tract infection  Ventilator-Associated Pneumonia  Central Line-Associated Bloodstream Infection      Low-Middle Income Countries |
| --- | --- |

**S 1 List of abbreviations**
