# Supplementary material for: Perceptions and attitudes of ICU physicians toward antibiotics prescribing and resistance: A cross-sectional study
Source: PLoS One. 2022 Sep 15;17(9):e0273673. doi: 10.1371/journal.pone.0273673 (PMC9477304; doi:10.1371/journal.pone.0273673)
Supplement: S2 File — (DOCX) [file pone.0273673.s005.docx]

**S2 File . Test-retest of the domains: consistency two way mixed single ICC.**

| **Domain** | **ICC** | **95% CI** |
| --- | --- | --- |
| **Domain 1 ^a^**  **Domain 2 ^b^**  **Domain 3 ^c^**  **Domain 4 ^d^**  **Domain 5 ^e^**  **Domain 6 ^f^** | **0.52**  **0.61**  **0.82**  **0.66**  **0.56**  **0.77** | **0.20-0.73**  **0.33-0.79**  **0.67-0.91**  **0.41-0.82**  **0.26-0.76**  **0.58-0.88** |

ICC: consistency two way mixed single intraclass correlation coefficient. CI: confidence interval.

^a^ Relevance as contributing factors to the development or spread of AMR: use of antibiotics. ^b^ Relevance as contributing factors to the development or spread of AMR: infection control measures. ^c^ Factors contributing to the spread of AMR. ^d^ Confidence in prescribing antibiotics. ^e^ Helpfulness of advice or computer-aided. ^f^ Helpfulness of implementation of antimicrobial stewardship measures.
