# Supplementary material for: Perceptions and attitudes of ICU physicians toward antibiotics prescribing and resistance: A cross-sectional study
Source: PLoS One. 2022 Sep 15;17(9):e0273673. doi: 10.1371/journal.pone.0273673 (PMC9477304; doi:10.1371/journal.pone.0273673)
Supplement: S4 File — (DOCX) [file pone.0273673.s007.docx]

**S4 File**

**Mean and standard deviation (SD) of each domain by surgeons’ professional profile and working setting (final sample).**

| **Variables** | **Domain 1 ^a^**  **mean (SD)** | **Domain 2 ^b^**  **mean (SD)** | **Domain 3 ^c^**  **mean (SD)** | **Domain 4 ^d^**  **mean (SD)** | **Domain 5 ^e^**  **mean (SD)** | **Domain 6 ^f^**  **mean (SD)** |
| --- | --- | --- | --- | --- | --- | --- |
| **Sex**  **Male**  **Female**  **P value** | **3.87 (1.66)**  **4.47 (1.79)**  **0.004** | **8.92 (3.20)**  **7.23 (2.62)**  **<0.001** | 4.04 (1.20)  4.21 (1.11)  0.232 | 12.18 (3.25)  12.15 (2.93)  0.933 | 9.91 (2.94)  9.42 (2.39)  0.135 | **8.23** (**2.58**)  **7.01** (**1.68**)  **<0.001** |
| **Years of experience**  **Less than 10 years**  **11 - 20 years**  **21 - 30 years**  **More than 30 years**  **P value** | 4.44 (1.79)  4.20 (1.71)  4.53 (1.76)  4.46 (1.94)  0.308 | 8.41 (3.10)  8.58 (2.95)  8.53 (3.10)  9.36 (3.65)  0.074 | 4.27 (1.17)  3.99 (1.16)  3.99 (1.29)  4.08 (1.12)  0.161 | **13.10** (**2.98**)  **11.91** (**3.26**)  **11.84** (**3.26**)  **12.12** (**3.12**)  **0.003** | 9.60 (2.90)  9.98 (2.92)  9.94 (2.90)  9.78 (2.75)  0.659 | 8.16 (2.70)  7.90 (2.47)  7.97 (2.46)  8.34 (2.45)  0.425 |
| **Type of hospital**  **University hospital**  **Community teaching hospital**  **Community hospital**  **P value** | 8.95 (3.19)  8.49 (3.21)  8.08 (2.96)  0.965 | **4.10** (**1.21**)  **3.93** (**1.11**)  **4.04** (**1.11**)  **0.002** | 12.14 (3.22)  12.21 (3.32)  12.29 (2.97)  0.417 | 10.05 (3.02)  9.38 (2.55)  9.90 (2.39)  0.985 | 8.25 (2.64)  7.74 (2.21)  7.96 (2.31)  0.025 | **8.24** (**2.64**)  **7.74** (**2.21**)  **7.96** (**2.31**)  **0.008** |
| **Hospital with antimicrobial stewardship team**  **Yes**  **No**  **Unsure**  **P value** | 4.41 (1.75)  4.24 (1.70)  4.84 (2.72)  0.265 | 8.62 (3.19)  9.07 (3.25)  7.68 (2.19)  0.094 | 4.06 (1.21)  4.06 (1.14)  4.28 (1.10)  0.657 | 12.26 (3.31)  11.78 (2.78)  12.92 (3.22)  0.148 | 9.72 (2.86)  10.15 (3.00)  10.36 (2.41)  0.207 | 8.09 (2.55)  7.97 (2.46)  7.84 (2.17)  0.801 |
| **Local guidelines for therapy of infections**  **Yes**  **No**  **Unsure**  **P value** | 4.47 (1.92)  4.15 (1.23)  4.00 (1.41)  0.154 | **8.62** (**3.12**)  **9.09** (**3.37**)  **6.40** (**2.01**)  **0.021** | 4.05 (1.21)  4.11 (1.12)  4.40 (1.17)  0.581 | 12.12 (3.20)  12.26 (3.08)  13.60 (4.79)  0.337 | **9.67** (**2.86**)  **10.50** (**2.88**)  **9.60** (**2.63**)  **0.010** | 8.05 (2.55)  8.10 (2.37)  7.80 (2.62)  0.925 |
| **Reports on local antibiotic resistance data**  **Yes**  **No**  **Unsure**  **P value** | 4.41 (1.77)  4.34 (1.89)  4.41 (1.40)  0.926 | 8.65 (3.15)  8.74 (3.34)  8.79 (2.60)  0.933 | 4.09 (1.24)  3.99 (1.05)  4.24 (1.18)  0.467 | 12.17 (3.22)  12.05 (3.14)  13.10 (3.35)  0.260 | 9.83 (2.85)  9.80 (2.88)  10.38 (3.18)  0.592 | 7.91 (2.53)  8.29 (2.48)  8.69 (2.36)  0.087 |

SD: standard deviation. ^a^ Relevance as contributing factors to the development or spread of AMR: use of antibiotics. ^b^ Relevance as contributing factors to the development or spread of AMR: infection control measures. ^c^ Factors contributing to the spread of AMR. ^d^ Confidence in prescribing antibiotics. ^e^ Helpfulness of advice or computer-aided. ^f^ Helpfulness of implementation of antimicrobial stewardship measures.
